# Association between sarcopenia and frailty in middle-aged and elder population: Findings from the China health and retirement longitudinal study

**DOI:** 10.7189/jogh.14.04163

**Published:** 2024-08-16

**Authors:** Yue Dong, Yuzhi Xi, Yahui Wang, Zhijun Chai

**Affiliations:** The First Affiliated Hospital of Soochow University, Suzhou, China

## Abstract

**Background:**

The relationship between sarcopenia and frailty among middle-aged and elder adults remains unclear. This study conducted a cross-sectional and longitudinal analysis to investigate the association of sarcopenia and frailty in the middle-aged and elder Chinese population.

**Methods:**

Our data were drawn from the China Health and Retirement Longitudinal Study. Sarcopenia status was assessed according to the Asian Working Group for Sarcopenia 2019 criteria and categorised into: no sarcopenia, possible sarcopenia, sarcopenia, and severe sarcopenia. A 38-item deficit-accumulation frailty index was constructed to assess frailty trajectories at each visit. Generalised linear regression models were performed to analyse the cross-sectional associations between sarcopenia and frailty index. The Group-based trajectory modelling was adopted to identify potential frailty trajectories, and we then examined the associations of sarcopenia and frailty trajectories using logistic regression analysis.

**Results:**

A total of 13 218 participants were enrolled in the cross-sectional analysis and 4200 individuals were included in the longitudinal study. The cross-sectional study found that possible sarcopenia (regression coefficient (β) = 0.76; 95% confidence interval (CI) = 0.64–0.87, *P* < 0.001), sarcopenia (β = 0.56; 95% CI = 0.37–0.75, *P* < 0.001) and severe sarcopenia (β = 1.35; 95% CI = 0.97–1.73, *P* < 0.001) were significantly associated with higher frailty index. The longitudinal study indicated that participants with possible sarcopenia (odds ratio (OR) = 2.46; 95% CI = 1.77–3.42, *P* < 0.001), sarcopenia (OR = 1.87; 95% CI = 1.27–2.74, *P* < 0.001) and severe sarcopenia (OR = 6.57; 95% CI = 3.14–13.77, *P* < 0.001) had a higher risk of accelerated progression of frailty compared to those with no sarcopenia.

**Conclusions:**

Possible sarcopenia, sarcopenia, and severe sarcopenia were associated with higher levels of frailty and accelerated progression of frailty. Therefore, clinical medical professionals should pay more attention to frailty status in individuals who have possible sarcopenia and sarcopenia.

Frailty has emerged as a substantial health challenge as the world's aging population grows [1,2]. Substantial evidence stated that frailty was associated with several adverse outcomes, including mortality, hospitalisation, and falls [3,4]. The currently operational definitions for frailty mainly contain two types: one defines frailty as the accumulation of deficits, and the other defines frailty as a physical phenotype [5]. Despite no consensus definition has been reached, frailty is considered as a dynamic process that worsens with aging [1]. Therefore, evaluating long-term trajectories of frailty and identifying potential risk factors could be essential for developing effective strategies for prevention [6,7].

Sarcopenia is a syndrome characterised by progressive and generalised loss of skeletal muscle mass, sharing a set of metabolic, hematologic, and inflammatory biomarkers with frailty [8–10]. The mechanisms underlying the association of sarcopenia and frailty remain unclear. Individuals with sarcopenia might have pathophysiological changes including muscle mitochondria dysfunction, oxidative stress, hyper-inflammation status, microvascular endothelial dysfunction, and multiple metabolic disorders, which could contribute to promoting frailty [8,11,12]. In addition, frailty and sarcopenia share many commonalities in the underlying mechanisms and pathophysiologic processes, like aging, hormonal imbalance, low physical activities, poor nutritional status, and comorbidities [13]. However, limited evidence focused on the association between sarcopenia and frailty in general middle-aged and elder adults, and only a few studies reported a significantly positive relationship between sarcopenia and frailty among middle-aged and elder adults with chronic disease [14,15]. A study pointed out that sarcopenia was an independent risk factor for frailty in elderly patients with chronic kidney disease [16]. Current studies on sarcopenia and frailty mainly reported on single-time assessment of frailty, studies on the association between sarcopenia and the progression of frailty are limited [16,17]. Moreover, few relevant studies have been conducted in the Asian region. There might be inconsistent results due to differences in the study populations. Previous studies highlighted that compared with Whites, Asians tend to have lower BMI, skeletal muscle mass, muscle strength, and fat mass, which emphasised the importance of conducting research on sarcopenia in different populations [16].

The elderly population in China is growing exponentially and this growth will last for decades. According to the data survey, there will be 400 million Chinese citizens aged over 65 and 150 million citizens aged over 80 by 2050, which is likely to bring a formidable health care challenge [18]. However, there is a paucity of studies focused on the association between sarcopenia and frailty in Chinese middle-aged and elder adults. Therefore, we aimed to explore the relationship between sarcopenia and frailty progression among middle-aged and elder residents based on the China Health and Retirement Longitudinal Study (CHARLS).

## METHODS

### Study design and population

Our data was drawn from the CHARLS, which is an ongoing nationally longitudinal investigation among the middle-aged and elder population in China [19]. CHARLS was established in 2011 (Wave 1), and the respondents were followed in 2013 (Wave 2), 2015 (Wave 3), 2018 (Wave 4), and 2020 (Wave 5). Briefly, CHARLS used face-to-face computer-assisted personal interviews and structured questionnaires to conduct the survey, and the collected information on: social demographics, lifestyle characteristics, and health-related information of the respondents. Previous studies reported a detailed description of the questionnaire [19,20].

This study consisted of a cross-sectional study and a longitudinal study. In the cross-sectional study, we used data from Wave 3 of CHARLS. A total of 21 095 subjects were interviewed in Wave 3. The exclusion criteria were as follows: (1) individuals without valid data of birth or age <40; (2) individuals with missing information of sarcopenia; (3) individuals without relevant data of frailty. In the cohort study, data were analysed from Wave 3 to Wave 5 of CHARLS. Wave 3 not Wave 1 was used as baseline because data from Wave 3 were relatively more complete, accurate, and updated. The cohort study further excluded: (1) subjects with missing values on frailty in Wave 4; (2) individuals with missing values on frailty in Wave 5.

### Assessment of sarcopenia status

Sarcopenia status was assessed according to the Asian Working Group for Sarcopenia 2019 (AWGS 2019) criteria and was divided into: possible sarcopenia, sarcopenia, and severe sarcopenia [21]. Possible sarcopenia was defined as the presence of low muscle strength or reduced physical performance. When low muscle mass with low muscle strength or low physical performance was observed, sarcopenia would be considered. Severe sarcopenia was referred to as the coexistence of low muscle strength, low muscle mass, and low physical performance.

Muscle strength was assessed by handgrip strength, which was measured under the guidance of trained examiners using dynamometers (Nantong Yuejian Physical Measurement Instrument Co., Ltd, Nantong, China). The dominant hand and non-dominant hand of each subject were measured twice, and the maximum value was viewed as the value of grip strength. The threshold points for low grip strength was <18 kg for females and <28 kg for males following the consensus from AWGS 2019 [21].

Appendicular skeletal muscle mass (ASM) was estimated by an anthropometric equation model for Chinese residents that has been validated by several studies [22,23].

*ASM* = 0.193 × *body weight* + 0.107 × *height* – 4.157 × *sex* – 0.037 × *age* – 2.631.

In this model, the body weight, height, and age were assessed in kilograms, centimetres, and years, respectively. For gender, 1 indicated male and 2 for female. The assessment of height-adjusted muscle mass (ASM/Ht^2^) was calculated by dividing the ASM by the square of the height in meters. Previous studies stated that low muscle mass was defined as the lowest 20% percentile ASM/Ht^2^ of the study population [24–26]. Therefore, low muscle mass was defined as the value of ASM/Ht^2^<5.45 kg/m^2^ in females and <7.10 kg/m^2^ in males.

Physical performance was assessed using gait speed and chair stand test. low physical performance was referred to as a 6-m walk <1.0 m/s or 5-time chair stand tests ≥12 seconds following the consensus from AWGS 2019 [21].

### Assessment of frailty

Frailty was assessed by the frailty index (FI), which was calculated as the accumulation of age-related health deficits [27]. We constructed FI following a standard procedure that was described previously [27,28]. A total of 38 items were selected to construct FI, including self-reported health status, comorbidity, physical function, depressive symptoms, and cognition (Table S1 in the **Online Supplementary Document**). The FI for each participant was calculated as the sum of present health deficits, and a higher score indicated a higher degree of frailty. Frailty trajectory was evaluated by the repeated measurements of FI. Therefore, we assessed the FI of the individuals at Wave 3, Wave 4, and Wave 5.

### Covariates

Covariates included social demographics and lifestyle characteristics assessed at baseline, which were obtained from Wave 3. Social demographics included age (in years), sex (male or female), body mass index (BMI) (in kg/m^2^), length of education (≥9 years, 6–9 years or ≤6 years), marital status (married or single), levels of income (<10 000 Chinese Yuan (Ұ) per year or ≥10 000 Ұ per year), employment status (yes or no) and medical insurance coverage (uninsured or public or private). Lifestyle characteristics included smoking status (yes or no), drinking habit (yes or no), and exercise status (frequent or infrequent).

### Statistical analyses

Descriptive analysis included means (±standard deviation (SD)) for continuous variables, and categorical variables were presented for frequencies (percentages). First, we used analysis of variance for continuous variables and χ^2^ tests for categorical variables to compare baseline characteristics between sarcopenia status in the cross-sectional study.

Second, generalised linear regression models were performed to analyse the associations between sarcopenia status and FI in the cross-sectional study, and regression coefficients (β) and 95% CI were calculated with no sarcopenia as the reference.

Third, the trajectories of FI were identified by group-based trajectory modelling (GBTM), which is an exploratory method used for classifying developmental trajectories in the population to identify subgroups that follow distinct trajectories over time [29]. The principle is to assume that there were several potential subgroups with different development trajectories or patterns in the population [30]. GBTM could merge trajectories and determine the form and number of groups that best fit the data [31,32]. Finding the most appropriate model was an iterative process. Several indicators could be used in model selection, including Bayesian information criterion (BIC), and the average posterior probability (AvePP), and the proportions of subgroups. A closer 0 of BIC, AvePP>0.7, and all proportions of subgroups >5% indicated a better model [30].

Fourthly, multivariate logistic regression analysis was used to estimate the relationship between sarcopenia status and different trajectories of FI. To control for potential confounding factors, we adjusted for age and sex in the first model. The second model was further adjusted for BMI, length of education, marital status, levels of income, employment status, medical insurance coverage, smoking status, drinking habit, and exercise status. The potential effect modification was evaluated using stratified analysis by age (<60 vs. ≥60 years), sex (male vs. female), BMI groups (non-obese: BMI<25 kg/m^2^ vs. obese: BMI≥25 kg/m^2^), smoking status (current non-smoker vs. current smoker) and drinking habit (current non-drinker vs. current drinker). Statistical analyses were performed using SAS version 9.4 (SAS Institute Inc., Cary, NC, USA) and Stata (Stata Corp., College Station, TX). Two-tailed *P-*value <0.05 was considered statistically significant.

## RESULTS

### Baseline characteristics of the study population in the cross-sectional and longitudinal study

**Figure 1:**
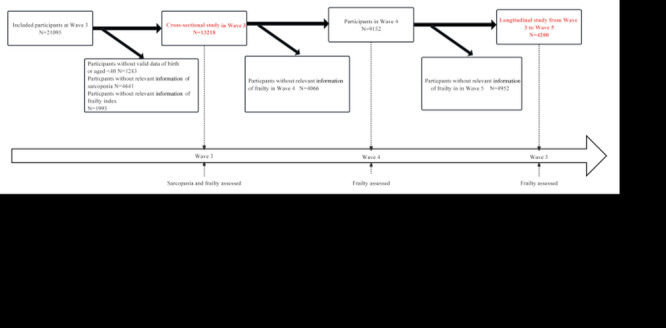
Timeline of the study design.

In the cross-sectional study, a total of 21 095 subjects were interviewed in Wave 3. According to the exclusion criteria mentioned above, we excluded 1243 individuals without valid data of birth, 4641 individuals lacked information on sarcopenia, and 1993 individuals without data on FI. Finally, the cross-sectional study consisted of 13 218 participants. In the cohort study, we further excluded subjects with missing values on frailty in Wave 4 (n = 4066) and Wave 5 (n = 4952), leaving 4200 participants for the cohort study. The detailed selection process is shown in **Figure 1**.

The baseline characteristics of the 13 218 participants grouped according to sarcopenia status in the cross-sectional study are listed in **Table 1**. All participants were categorised into four groups: no sarcopenia (n = 10 274), possible sarcopenia (n = 2035), sarcopenia (n = 730), and severe sarcopenia (n = 179). In contrast to those with no sarcopenia, participants with severe sarcopenia were older and more likely to be female. The characteristics of the 4200 participants included in the longitudinal study are presented in Table S2 in the **Online Supplementary Document**. The mean age was 57.50 years, and 2086 (49.67%) individuals were females.

**Table 1:** Baseline characteristics of all participants by sarcopenia status in the cross-sectional study

Variables	Total	No sarcopenia	Possible sarcopenia	Sarcopenia	Severe sarcopenia	*P*-value
	**n = 13 218, 100%**	**n = 10 274, 77.73%**	**n = 2035, 15.40%**	**n = 730, 5.5%**	**n = 179, 1.35%**	
Demographics, age, (mean ± SD)	58.81 ± 9.69	57.16 ± 8.87	62.24 ± 9.75	68.98 ± 9.43	72.91 ± 8.09	<0.0001
Gender, n (%)						<0.0001
*Male*	6404 (48.45)	5106 (49.70)	881 (43.29)	337 (46.16)	80 (44.69)	
*Female*	6814 (51.55)	5168 (50.30)	1154 (56.71)	393 (53.84)	99 (55.31)	
*BMI, (mean ± SD)*	24.06 ± 3.95	24.17 ± 3.84	25.53 ± 3.78	19.63 ± 1.89	19.62 ± 2.03	<0.0001
Marital status, n (%)						<0.0001
*Married*	11 847 (89.63)	9453 (92.01)	1733 (85.16)	542 (74.25)	119 (66.48)	
*Single*	1371 (10.37)	821 (7.99)	302 (14.84)	188 (25.75)	60 (33.52)	
Length of education, n (%)						<0.0001
*≤6 years*	7969 (64.63)	5852 (60.70)	1425 (75.68)	567 (85.91)	125 (85.62)	
*6–9 years*	2884 (23.39)	2476 (25.68)	319 (16.94)	73 (11.06)	16 (10.96)	
*≥9 years*	1477 (11.98)	1313 (13.62)	139 (7.38)	20 (3.03)	5 (3.42)	
Individual income, n (%)						<0.0001
*<10 000￥ per year*	11 332 (85.87)	8567 (83.47)	1883 (92.80)	707 (97.12)	175 (99.43)	
*≥10 000￥ per year*	1865 (14.13)	1697 (16.53)	146 (7.20)	21 (2.88)	1 (0.57)	
Employment status, n (%)						0.04
*Unemployed*	519 (3.93)	380 (3.70)	103 (5.06)	29 (3.97)	7 (3.93)	
*Employed*	12 694 (96.07)	9891 (96.30)	1931 (94.94)	701 (96.03)	171 (96.07)	
Medical insurance coverage, n (%)						<0.0001
*Uninsured*	899 (6.87)	671 (6.59)	147 (7.34)	65 (9.04)	16 (8.99)	
*Public*	11 775 (89.98)	9153 (89.87)	1815 (90.57)	647 (89.99)	160 (89.89)	
*Private*	412 (3.15)	361 (3.54)	42 (2.10)	7 (0.97)	2 (1.12)	
**Lifestyle characteristics**						
Smoking status, n (%)						<0.0001
*Current non-smoker*	9431 (71.38)	7209 (70.18)	1581 (77.81)	499 (68.36)	142 (79.33)	
*Current smoker*	3782 (28.62)	3063 (29.82)	451 (22.19)	231 (31.64)	37 (20.67)	
Drinking status, n (%)						<0.0001
*Current non-drinker*	8314 (62.92)	6226 (60.62)	1446 (71.09)	503 (68.90)	139 (77.65)	
*Current drinker*	4899 (37.08)	4044 (39.38)	588 (28.91)	227 (31.10)	40 (22.35)	
Physical exercise, n (%)						<0.0001
*Infrequent exerciser*	8612 (65.15)	6554 (63.79)	1420 (69.78)	504 (69.04)	134 (74.86)	
*Frequent exerciser*	4606 (34.85)	3720 (36.21)	615 (30.22)	226 (30.96)	45 (25.14)	

BMI – body mass index, SD – standard deviation, ￥ – Chinese Yuan

### Cross-sectional associations of sarcopenia status with frailty

The associations of sarcopenia status with FI at baseline are presented in Table S3 in the **Online Supplementary Document**. After adjustment for all covariates, possible sarcopenia (β = 0.76; 95% CI = 0.64–0.87, *P* < 0.001), sarcopenia (β = 0.56; 95% CI = 0.37–0.75, *P* < 0.001) and severe sarcopenia (β = 1.35; 95% CI = 0.97–1.73, *P* < 0.001) were significantly associated with higher FI.

### Frailty trajectory modelling and longitudinal association of sarcopenia status with frailty progression

**Figure 2:**
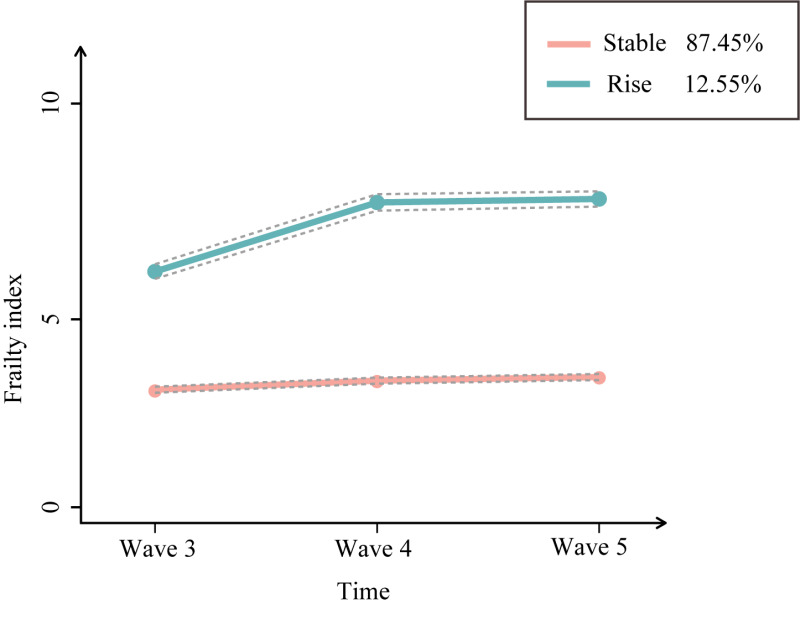
Trajectories of the frailty index. The solid lines mean estimated values, and the dotted lines display the 95% confidence interval.

Detailed information on the model fitting process is shown in **Table 2**. We found that Group 2 was the best fitting model. Group 2 had the smallest value of BIC, and the AvePP values were higher than 0.7. All the proportions of subgroups within the group were >5%. Therefore, two groups of frailty trajectories were identified (**Figure 2**). The stable group had a low level of FI at baseline and then maintained a stable trend. And rapidly rising group was characterised by the fact that FI at baseline was higher and kept accelerating. The ascending trend was obvious in the rising group, and the FI ascended from 6.30 to 8.32.

**Table 2:** AvePP of group assignment and BIC statistics of model fit

Number	BIC	Proportion, %	AvePP
1	−28549.86	100.00	100.00
2	−26911.62	87.45/12.55	98.43/90.55
3	−26922.17	78.27/17.97/3.76	95.89/84.86/91.82
4	−26940.34	87.10/12.90	98.39/90.66
5	−26939.99	87.10/12.90	98.35/90.85
6	−26913.83	87.25/12.75	98.39/90.74

AvePP – Average posterior probability BIC – Bayesian Information Criterion

A total of 527 individuals presented the rapidly rising trajectory of FI. The associations of sarcopenia status and frailty progression are listed in the **Table 3**. After adjustment of all covariates, we found that participants with possible sarcopenia (OR = 2.46; 95% CI = 1.77–3.42, *P* < 0.001), sarcopenia (OR = 1.87; 95% CI = 1.27–2.74, *P* < 0.001) and severe sarcopenia (OR = 6.57; 95% CI = 3.14–13.77, *P* < 0.001) were related to the accelerated progression of frailty in comparison with individuals with no sarcopenia.

**Table 3:** Associations of sarcopenia status and frailty progression in the longitudinal study

	OR	95% CI	*P*-value
Model 1*			
No sarcopenia	Reference	Reference	NA
Possible sarcopenia	2.60	1.89−3.57	<0.0001
Sarcopenia	1.69	1.17−2.44	<0.0001
Severe sarcopenia	5.06	2.50−10.23	<0.0001
**Model 2†**			
No sarcopenia	Reference	Reference	NA
Possible sarcopenia	2.46	1.77−3.42	<0.0001
Sarcopenia	1.87	1.27−2.74	<0.0001
Severe sarcopenia	6.57	3.14−13.77	<0.0001

CI – confidence interval, NA – not available, OR – odds ratio

*Model 1 adjusted for age and sex.

†Model 2 adjusted for age, sex, body mass index (BMI), education, marriage, income, employment, insurance, smoking, drinking, and exercise.

### Subgroup analysis

**Figure 3:**
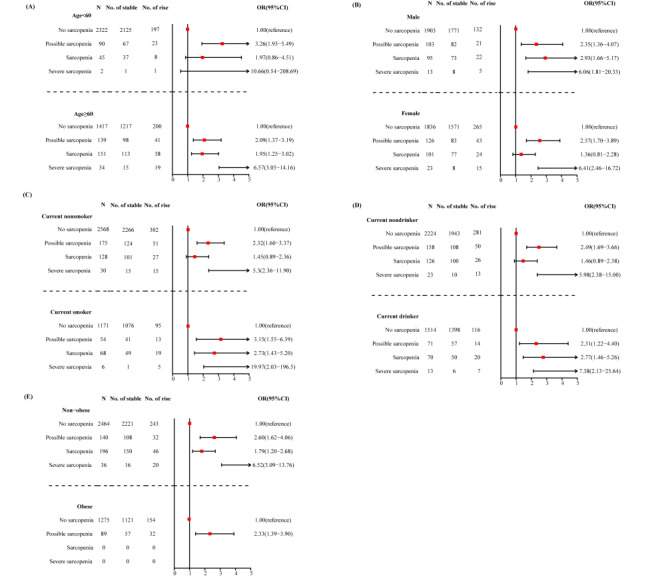
Subgroup analyses of association between sarcopenia status and different trajectories of frailty index by age, sex, BMI, smoking status and drinking habit. **Panel A.** Analyses of sarcopenia and trajectories of frailty index stratified by age. **Panel B.** Analyses of sarcopenia and trajectories of frailty index stratified by sex. **Panel C.** Analyses of sarcopenia and trajectories of frailty index stratified by smoking status. **Panel D.** Analyses of sarcopenia and trajectories of frailty index stratified by drinking habit. **Panel E.** Analyses of sarcopenia and trajectories of frailty index stratified by BMI. BMI – body mass index

We performed stratified analysis by age (<60 vs. ≥60 years), sex (male vs. female), BMI groups (non-obese: BMI<25 kg/m^2^ vs. obese: BMI≥25 kg/m^2^), smoking status (current non-smoker vs. current smoker) and drinking habit (current non-drinker vs. current drinker). Subgroup analysis of sex, BMI, smoking status, and drinking habit all showed that severe sarcopenia might related to an ascending trend of frailty (**Figure 3**, panels B, C, D, E). Meanwhile, we observed no significant relationship between severe sarcopenia and frailty progression among participants under 60 (**Figure 3**, panel A).

## DISCUSSION

Based on data from CHARLS, we explored the relationship between sarcopenia status and frailty progression among middle-aged and elder adults in a real-world setting. Our results revealed that possible sarcopenia, sarcopenia, and severe sarcopenia were independently and positively associated with higher levels of frailty in the cross-sectional study. In the cohort study, we measured FI three times to identify the trajectories of frailty progression. The results showed that possible sarcopenia, sarcopenia, and severe sarcopenia were all positively related to ascending trend of frailty. Specifically, individuals with severe sarcopenia had a higher risk of frailty progression than those with sarcopenia and possible sarcopenia. In addition, we found severe sarcopenia might be related to an ascending trend of frailty in the stratified analysis of sex, BMI, smoking status, and drinking habits. However, no significant association between severe sarcopenia and frailty progression among participants under 60 was observed.

The relationship between sarcopenia and frailty among middle-aged and elder adults has been poorly studied. Our findings demonstrated that middle-aged and elder adults with sarcopenia had higher levels of FI compared to those with no sarcopenia. Current studies focused on sarcopenia and frailty from other countries are limited, and most of these data have explored the relevance of sarcopenia and frailty in patients with chronic disease [33–35]. Only a multicentre prospective observational study from Spain reported that sarcopenia and frailty were frequent and interrelated conditions in polypathological patients [17], which was consistent with our results. A study from 29 Chinese clinical centres also reported that sarcopenia was positively associated with frailty in elderly patients with chronic kidney disease [16].

Our cohort study stated that sarcopenia was a warning sign of accelerated progression of frailty. The relation between sarcopenia and frailty was reported in most observational studies based on a single-time assessment of frailty [16,17]. Limited evidence took multiple measurements of frailty to observe the trajectory of frailty and examined the association between sarcopenia and the progression of frailty. Previous studies highlighted that frailty was deemed a dynamic process that worsens with aging [1]. A single assessment of frailty could not reflect the subsequent progression of frailty. Therefore, continuously observing the frailty in elder and middle-aged adults and evaluating long-term frailty trajectories could be significant for developing effective prevention strategies. Data from our study has helped to fill the gap to some extent.

We assessed the association between possible sarcopenia, according to the AWGS 2019 algorithm, and the progression of frailty. Although no study has reported the association between possible sarcopenia and frailty, several studies have assessed the relationships between possible sarcopenia and frailty components such as cognition and depression [36,37]. The current study demonstrated that elderly individuals with possible sarcopenia had a higher risk of mild cognitive impairment compared to those with no sarcopenia [36]. A longitudinal study also indicated a persistently higher risk of depression in the participants developed who possible sarcopenia [37].

Our findings indicated that adults with severe sarcopenia had higher risks of frailty progression in contrast to those with sarcopenia and possible sarcopenia. Severe sarcopenia is defined as the presence of low muscle mass, low muscle strength, and low physical performance [21]. Sarcopenia was considered as low muscle mass with low muscle strength or low physical performance and possible sarcopenia is defined as low muscle strength or reduced physical performance [21]. Therefore, the disease progression of severe sarcopenia is further and the relationship between severe sarcopenia and progression of frailty might be stronger.

We observed no significant associations between severe sarcopenia and progression of frailty among the participants under 60. The lack of association may be due to the low proportion of participants under 60 with severe sarcopenia in the study (0.05%), giving insufficient power to detect meaningful differences [38].

We believed that this study was one of the few studies conducted on sarcopenia and frailty among elder and middle-aged population from China. However, there are several limitations. First, our cohort study included subjects who had attended Wave 3, Wave 4 and Wave 5 might have selection bias because relatively more smokers (28.62 vs. 30.93%, **Table 1**, Table S2 in the **Online Supplementary Document**) and drinkers (37.08 vs. 39.72%, **Table 1**, Table S2 in the **Online Supplementary Document**) were included compared with participants at baseline. Second, because several deficits included in the FI calculation were self-reported, there might exist inevitably a potential for information bias. Third, this study could only identify the association between sarcopenia and frailty. Therefore, long-term cohort studies are needed to further investigate the relationship between sarcopenia and frailty, including underlying biological mechanisms. In addition, clinical randomised controlled trials should be conducted to evaluate the effectiveness of different intervention strategies for sarcopenia and frailty.

## CONCLUSIONS

In conclusion, this study assessed the cross-sectional relationship between sarcopenia and frailty and longitudinal associations of sarcopenia with the progression of frailty in Chinese middle-aged and elderly adults. Our findings indicated that individuals with possible sarcopenia, sarcopenia, and severe sarcopenia had higher levels of frailty and higher risks of rapid frailty progression compared to those without sarcopenia. The results of our study might be significant for developing effective strategies for preventing frailty and promoting healthy aging for middle-aged and elder adults. We suggested that clinical medical professionals should pay more attention to frailty in middle-aged and elder adults with possible sarcopenia, sarcopenia, and severe sarcopenia. Appropriate interventions such as nutritional supplementation, exercise, and physical therapy are needed to slow and prevent the progression of frailty [39,40]. Moreover, clinic diagnosis tools and operational management guidelines for frailty should be explored to improve the effectiveness of interventions.

## Additional material


Online Supplementary Document
