# Supplementary material for: Association between sarcopenia and frailty in middle-aged and elder population: Findings from the China health and retirement longitudinal study
Source: J Glob Health. 2024 Aug 16;14:04163. doi: 10.7189/jogh.14.04163 (PMC11327846; doi:10.7189/jogh.14.04163)
Supplement: Online Supplementary Document [file jogh-14-04163-s001.pdf]

Table S1. The 38 items used to construct the frailty index

No.	Description of the items	Cut-off value
1	Self-reported general health status.	Poor or fair = 1, excellent, very good, or good = 0
2	Self-reported physician diagnosed hypertension.	Yes = 1, No = 0
3	Self-reported physician diagnosed diabetes.	Yes = 1, No = 0
4	Self-reported physician diagnosed dyslipidemia.	
5	Self-reported physician diagnosed stroke.	Yes = 1, No = 0
6	Self-reported physician diagnosed cancer.	Yes = 1, No = 0
7	Self-reported physician diagnosed asthma.	Yes = 1, No = 0
8	Self-reported physician diagnosed any emotional, nervous, or psychiatric problems.	Yes = 1, No = 0
9	Self-reported physician diagnosed memory-related disease. *	Yes = 1, No = 0
10	Difficulty with managing money.	Yes = 1, No = 0
11	Difficulty with taking medications.	Yes = 1, No = 0
12	Difficulty with shopping for groceries.	Yes = 1, No = 0
13	Difficulty with preparing meals.	Yes = 1, No = 0
14	Difficulty with doing housework.	Yes = 1, No = 0
15	have any difficulties with making phone calls.	Yes = 1, No = 0
16	I was bothered by things that don’t usually bother me.	Yes = 1, No = 0
17	I had trouble keeping my mind on what I was doing.	Yes = 1, No = 0
18	Feeling depressed much of time.	Yes = 1, No = 0
19	Feeling that everything is an effort much of	Yes = 1, No = 0

	time.	
20	I felt hopeful about the future.	Yes = 1, No = 0
21	I felt fearful.	Yes = 1, No = 0
22	My sleep was restless.	Yes = 1, No = 0
23	Feeling happy much of time.	Yes = 1, No = 0
24	Feeling lonely much of time.	Yes = 1, No = 0
25	Feeling that could not get going much of time.	Yes = 1, No = 0
38	Cognition: (memory test score + orientation test score) / 13 †	Continuous, ranging from 0 to 1

* Memory-related disease indicates Alzheimer's disease or dementia, organic brain senility, or other serious memory impairment.

† Cognition: (memory test score + orientation test score) / 13 Continuous, ranging from 0 to 1. The memory score is the average of words that are not recalled in the immediate and delayed word recall tasks. The orientation test comprises three questions about the day of the date of the month, and the year.

Table S2 Baseline characteristics of all participants by frailty trajectory groups in the longitudinal study

	Total<br>N=4200<br>100%	Stable<br>N=3673<br>87.45%	Rise<br>N=527<br>12.55%	*P*-value
**Social demographics**				
Age, (mean ± SD)	57.50 ± 9.05	56.96 ± 8.87	61.25 ± 9.39	$P < 0.0001$
Gender, n (%)				$P < 0.0001$
Male	2114(50.33%)	1934(52.65%)	180(34.16%)	
Female	2086(49.67%)	1739(47.35%)	347(65.84%)	
Marital status, n (%)				$P < 0.0001$
Married	3854(91.76%)	3412(92.89%)	442(83.87%)	
Single	346(8.24%)	261(7.11%)	85(16.13%)	
Length of education, n (%)				$P < 0.0001$
≤ 6 years	2658(63.29%)	2240(60.99%)	418(79.32%)	
6-9 years	1078(25.67%)	995(27.09%)	83(15.75%)	
≥ 9 years	464(11.05%)	438(11.92%)	26(4.93%)	
Individual income, n (%)				$P < 0.0001$
Less than 10000￥ per year	3583(85.35%)	3077(83.82%)	506(96.02%)	
More than 10000￥ per year	615(14.65%)	594(16.18%)	21(3.98%)	
Employment status, n (%)				$P = 0.63$
Unemployed	134(3.19%)	119(3.24%)	15(2.85%)	
Employed	4066(96.81%)	3554(96.76%)	512(97.15%)	
Medical insurance, n (%)				$P = 0.10$
Uninsured	274(6.59%)	239(6.57%)	35(6.73%)	
Public	3746(90.11%)	3270(89.91%)	476(91.54%)	
Private	137(3.30%)	128(3.52%)	9(1.73%)	
BMI, (mean ± SD)	23.66 ± 3.86	23.61 ± 3.61	24.01 ± 5.26	$P = 0.03$
**Lifestyle characteristics**				
Smoking status, n (%)				$P = 0.00$
Current non-smoker	2901(69.07%)	2506(68.23%)	395(74.95%)	
Current smoker	1299(30.93%)	1167(31.77%)	132(25.05%)	
Drinking status, n (%)				$P < 0.0001$
Current non-drinker	2531(60.28%)	2161(58.85%)	370(70.21%)	
Current drinker	1668(39.72%)	1511(41.15%)	157(29.79%)	
Physical exercise, n (%)				$P = 0.27$
Frequent exerciser	2666(63.48%)	2320(63.16%)	346(65.65%)	
Infrequent exerciser	1534(36.52%)	1353(36.84%)	181(34.35%)	
FI at Wave 3, (mean ± SD)	3.50 ± 2.06	3.09 ± 1.52	6.30 ± 2.97	$P < 0.0001$
FI at Wave 4, (mean ± SD)	3.95 ± 2.43	3.34 ± 1.62	8.20 ± 2.81	$P < 0.0001$
FI at Wave 5, (mean ± SD)	4.05 ± 2.48	3.43 ± 1.72	8.32 ± 2.70	$P < 0.0001$

FI – frailty index

Table S3 Association of sarcopenia status with frailty in the baseline

	β	95% CI	*P*-value
**Model 1***			
No sarcopenia	Reference	Reference	NA
Possible sarcopenia	0.87	0.75-0.98	$P < 0.0001$
Sarcopenia	0.60	0.42-0.78	$P < 0.0001$
Severe sarcopenia	1.23	0.88-1.57	$P < 0.0001$
**Model 2 †**			
No sarcopenia	Reference	Reference	NA
Possible sarcopenia	0.76	0.64-0.87	$P < 0.0001$
Sarcopenia	0.56	0.37-0.75	$P < 0.0001$
Severe sarcopenia	1.35	0.97-1.73	$P < 0.0001$

β – regression coefficients; 95% CI – 95% confidence interval, NA – not available

*Model 1 adjusted for age and sex.

†Model 2 adjusted for age, sex, BMI, education, marriage, income, employment, insurance, smoking, drinking, and exercise.
